# An integrated conceptual framework for evaluating and improving ‘understanding’ in informed consent

**DOI:** 10.1186/s13063-017-2204-0

**Published:** 2017-10-17

**Authors:** Sabine Bossert, Daniel Strech

**Affiliations:** Institute for History, Ethics and Philosophy of Medicine, Hannover Medical School, Carl-Neuberg-Str. 1, 30625 Hannover, Germany

**Keywords:** Informed consent, Participatory consent improvement, Understanding in informed consent

## Abstract

**Background:**

The development of understandable informed consent (IC) documents has proven to be one of the most important challenges in research with humans as well as in healthcare settings. Therefore, evaluating and improving understanding has been of increasing interest for empirical research on IC. However, several conceptual and practical challenges for the development of understandable IC documents remain unresolved.

**Methods:**

In this paper, we will outline and systematize some of these challenges. On the basis of our own experiences in empirical user testing of IC documents as well as the relevant literature on understanding in IC, we propose an integrated conceptual model for the development of understandable IC documents.

**Results:**

The proposed conceptual model integrates different methods for the participatory improvement of written information, including IC, as well as quantitative methods for measuring understanding in IC.

**Conclusions:**

In most IC processes, understandable written information is an important prerequisite for valid IC. To improve the quality of IC documents, a conceptual model for participatory procedures of testing, revising, and retesting can be applied. However, the model presented in this paper needs further theoretical and empirical elaboration and clarification of several conceptual and practical challenges.

## Background

Informed consent (IC) is an important ethical and legal requirement in research with humans as well as in clinical care [1–3]. The Council for International Organizations of Medical Sciences, for example, specifies that valid IC can only be given “*after providing relevant information about the research and ascertaining that the potential participant has adequate understanding of the material facts*” [2]. Only if the ‘understanding’ requirement is met can IC be considered an effective means to protect participants’ and patients’ autonomy, and to maintain public trust [4–6].

Thus, evaluating and improving understanding has been of interest for empirical research on IC for several years [7–9]. However, there seems to be no agreed definition of the term ‘understanding’, which makes it difficult to compare the results of different studies [10, 11]. Further, various methods have been applied to test, improve, and sometimes retest understanding in IC. As we will argue below, different methods should be combined rather than applied independently to develop understandable IC documents.

## Methods

In this paper, we will first argue that it is necessary to clarify the meaning of ‘understanding’ in IC, and propose a working definition of the concept by distinguishing (the measurement of) ‘subjective’ and ‘objective’ understanding. Secondly, based on the relevant literature as well as own experience with participatory improvement of IC documents, we aim to systematize a set of methodological issues for the development of understandable IC documents. Thereby, we will focus on the engagement of members of the target group in this process. Thirdly, we will propose an integrated conceptual model for testing, improving and retesting IC documents. Finally, we shall outline core issues for future research on IC development.

## Analysis: conceptual and practical challenges for the development of understandable consent documents

In this section we outline five methodological and conceptual issues for testing and improving understanding in IC. These are (1) a clarification of the concept of ‘understanding’; (2) explaining the relationship between different approaches to empirical research on understanding in IC; (3) methodological challenges for the actual revision of IC documents; (4) conceptualizing benefits of involving members of the target population in the evaluation and revision of IC documents; and (5) the identification and recruitment of members of the target population for participatory consent improvement.

### Clarification of concepts: ‘subjective’ vs. ‘objective’ understanding and general understandability

The first issue for the evaluation and enhancement of ‘understanding’ in IC is to clarify what exactly is to be evaluated. The lack of an agreed definition of understanding in empirical consent research has been criticized before [12, 13]. The comparison of different studies assessing understanding requires a common notion of what understanding actually entails, as well as the application of comparable methods [10, 11]. In a systematic review of studies measuring understanding, Sand et al. [11] show that several distinct concepts are covered by the term ‘understanding’. Despite their varying conceptualizations, all the studies reviewed by Sand et al. [11] aim to measure understanding objectively, i.e., by testing participants’ knowledge or memory of certain facts. These studies do not generally evaluate how well the participants (subjectively) felt themselves to have understood the IC document.

For valid IC, however, IC documents should address prospective research participants’ needs and demands. Therefore, to improve IC documents it can be helpful to first identify text passages that are intrinsically hard to understand. Thus, it is also important to assess readers’ own perception of the documents, e.g., whether they feel that they (subjectively) understood the given information, and whether this information is adequate to come to an autonomous decision on their participation.

Finally, in some cases, it can be advantageous to assess general understandability instead of or in addition to objective/subjective understanding. If, for example, there are no members of an IC document’s target population available for testing, third parties can be asked to anticipate how difficult it would be for the target population at large to understand the given pieces of information. Additionally, members of the target population themselves could be asked how difficult they think it would be for others to understand the given information (general understandability). This information could help to generalize or contrast data on objective and subjective understanding. For example, if test readers are better educated or more experienced than average members of the target population, results on objective as well as subjective understanding would be biased (higher scores for both concepts of understanding compared to the average reader). However, such test readers could possibly anticipate that it is more difficult for less educated and experienced persons to understand the information given in the tested IC documents. Then, assessing general understandability could substantially complement data on subjective and objective understanding.

In line with this notion, we propose a working definition for three different types of ‘understanding’ and their conceptual relationship, which is explained in Table 1. All three types of understanding need to be distinguished from related concepts like comprehension, knowledge, or memory, terms that are frequently interchanged. According to our working definition, comprehension can be used as a synonym for understanding. We define ‘knowledge’ as “*facts, information, and skills acquired through experience or education*”. In other words, we presume readers of IC documents to gain some knowledge about certain facts as a consequence of having understood these facts. Whether or not they are able to recall these facts later from their memories is a question that needs to be addressed separately from the concept of understanding. However, according to our definition, the mentioned concepts are interrelated – understanding is one prerequisite of gaining knowledge, which is one perquisite of memorizing certain pieces of information later on. Of course, this definition is contestable and needs further consideration to be able to guide future research on understanding in IC. Irrespective of the nature of the definition used, we believe that it is essential to clarify the concept of ‘understanding’ before testing and revising IC documents.

**Table 1:** Different concepts of ‘understanding’

Types of ‘understanding’	Description	Exemplary methods of assessment
Objective understanding	Correct knowledge of certain facts after having read IC documents	Knowledge or memory tests by means of (1) standardized questionnaires or interviews, or (2) by asking participants to rephrase facts in their own words
Subjective understanding	Subjective impression of having understood certain facts after reading IC documents	Open question, e.g., “Do you think you have understood the given information? If not, what were the text passages you find difficult to understand?”
General understandability	Personal impression of whether the given information is in principle easy to understand for others, e.g., members of the target group	Open question, e.g., “How easy do you think will it be for others to understand the given information?”

### Complementary types of empirical research on understanding in IC

The three types of understanding also help to distinguish two complementary types of empirical research on understanding. First, there is the relatively established field of mainly quantitative studies measuring objective understanding [8], e.g., whether study participants understand the purpose and the risks of the research explained in the IC documents, whether they actually grasp the meaning of randomization, or whether they understand that their participation is voluntary and that they have the right to withdraw. These studies mostly use standardized questionnaires or interview surveys; many even conduct randomized controlled trials to systematically compare participants’ understanding after different interventions supporting the consent process [7, 9, 12, 14]. One prominent model for systematically assessing objective understanding is the Brief Informed Consent Evaluation Protocol, developed by Sugarman et al. [15], which uses a short questionnaire for telephone interviews to evaluate the quality of IC for clinical research.

More recently, a second field of research on understanding written information has been explored. The studies in this field mostly apply an iteration of mixed methods – standardized questionnaires, semi-structured individual interviews, focus groups – to test, revise and retest written information (e.g., [16, 17]). These so-called ‘user testings’ address all three types of ‘understanding’ – most user testings assess participants’ objective understanding and how easy it is for them to find certain pieces of information by means of standardized questionnaires. Many complement the standardized assessment of objective understanding by focusing on test readers’ ‘subjective understanding’, e.g., to what extent they themselves feel that they understood the given information (e.g., [18]), and some ask for test readers’ assessment of the documents’ general ‘understandability’ (e.g., [19]). In addition, user testings often aim to identify reasons for problems of understanding by means of qualitative interviews or focus group discussions [16, 18, 19]. Usually, they also revise the tested information documents according to the test readers’ feedback and retest the revised version [16, 17]. Some user testing studies also evaluate the revised documents against the original version by means of a randomized controlled trial, using quantitative methods [16]. The method was originally invented in Australia for testing product information leaflets [20]. Since then, user testings (and similar methods under different labels) have been applied to a growing variety of written information on various topics [21–24], including patient information documents for clinical trials (e.g., [16, 25, 26]).

Most endeavors of participatory improvement of IC documents for clinical care or research in humans use individual standardized and semi-structured interviews. For some evaluations of written health information and decision aids focus group interviews are conducted to identify test readers’ perceptions of a given document and the information it provides, including emotional responses and misunderstandings of certain issues [18, 19]. Compared to individual interviews, focus groups have some advantages for testing and improving written information – they allow participants to comment on the statements of others, to clear up misunderstandings amongst themselves, and to discuss complex and divisive issues. This allows the assessment of the relative relevance of different feedback, e.g., when participants put into perspective their own feedback after comments by other participants – and to identify contrasting views as well as the underlying rationales, e.g., when participants discuss certain issues amongst themselves and give reasons for their differing opinions. These insights from focus groups can make it easier to understand test readers’ opinions of the tested documents and their potential problems of understanding.

Both standardized assessments of understanding and the different methods for participatory testing and improving written information have specific strengths and weaknesses. While quantitative methods allow for a valid assessment of objective understanding and produce generalizable results, user testings and other participatory methods can help to identify reasons for problems of understanding. In addition, test readers’ own perceptions of the IC documents can be assessed for subjective understanding or emotional responses to certain pieces of information [19]. According to specific needs, IC evaluation might combine different methods to test, improve and retest high quality IC documents.

### Challenges for the systematic and transparent revision of IC documents after user testing

While methods for the assessment of objective and subjective understanding seem to be increasingly established, a remaining major challenge is the question of how to systematically and transparently revise the evaluated IC documents according to test readers’ feedback. On the basis of our own experience with the participatory evaluation and improvement of IC documents for biobank research, we identified several practical challenges in dealing with different types of feedback. These challenges are listed and further described in Table 2.

**Table 2:** Practical challenges for the systematic and transparent revision of informed consent documents

Challenges	Questions to answer for the revision of IC documents
Dealing with feedback or suggestions from different numbers of participants	Are suggestions expressed repeatedly by multiple individuals in different interviews or focus groups more important than suggestions made by just one test reader?
Dealing with participants’ conflicting opinions on the same topic	How can test readers’ conflicting opinions be addressed, e.g., if some participants think sub-headlines should be formulated as questions, while others prefer declarative sentences? Which suggestion should be used when conflicting opinions about the same topic have been expressed?
Trade-off between different reasonable suggestions	How can different well-reasoned but irreconcilable suggestions be addressed, e.g., if participants on the one hand suggest to abbreviate the whole text to make it more readable to everybody, while on the other hand, they want some topics to be explained in more detail or they ask for additional pieces of information?
Dealing with feedback or suggestions that do not seem reasonable to the authors	How should suggestions be handled that do not seem reasonable, i.e., that would not seem to increase objective understanding, or do not assist prospective research participants with their autonomous decision, e.g., when a particular test reader is interested in more background information on some rather marginal topic? And how can one systematically decide which suggestions are reasonable and which are not?
Making changes transparent and replicable	How can revisions in general and decisions in the abovementioned cases in particular be made transparent and accountable to others?

Faced with these challenges, it can be difficult to ensure that the improved IC documents truly address test readers’ feedback and that revisions are not primarily based on the authors’ interpretations and personal tastes. As a first step to increase the transparency and accountability of revisions, in our own focus group study we first grouped the statements of all test readers into categories according to the subject they dealt with. To reduce the number of statements in each category, we then combined consensual and contesting comments on each issue into different sub-categories. For the actual revision of the tested documents, we assigned a distinct code to each sub-category and noted, for each change made to the original document, to which piece of feedback it referred. Additionally, we documented how we addressed each piece of feedback. When we did not address a particular suggestion we also gave reasons for our decision. All decisions regarding revisions were discussed by members of our research group to avoid bias in interpretation or revision. This method is one possible approach to increasing transparency and accountability, but needs further development and refinement.

Authors of previous user testings identify three general sources for their changes to the original documents, namely (1) feedback from the user testing, (2) best-practice guidelines in information wording and clear writing, and (3) authors’ experiences with writing patient information documents [16, 23, 25, 26]. However, at present, to our knowledge, no one has reported how exactly revisions were made based on test readers’ feedback and how they dealt with the challenges outlined in Table 2. If feedback from user testings is considered only one of three sources for the revision of the original document, what is the actual contribution of the target population to the development and improvement of IC documents?

### Why members of the target population should be involved in the validation and improvement of IC documents

**Fig. 1:**
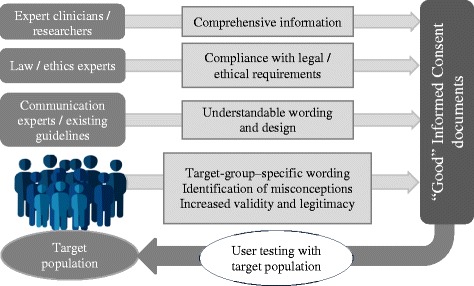
Multidisciplinary development of informed consent documents

Figure 1 shows different actors and their roles in developing IC documents. In order to enable autonomous consent decisions, prospective research participants need to obtain all the relevant information about the purpose, process, risks, and benefits of the research project they are asked to consent to [27, 28]. This information can best be given by researchers or medical experts. Therefore, these groups need to be included in the development of IC documents to ensure the correctness and comprehensiveness of given information. Authors of IC documents should then make sure that they meet all relevant ethical and legal norms. To fulfil this requirement, ethicists and law experts can be included in the process of writing and validating IC documents. Finally, for the IC documents to be truly informative, the given information must be easy to understand for members of the target population, and be adequately well presented. This can be ensured by professional writers or communications experts. Additionally, the application of existing guidelines for information wording and design or clear writing, e.g., the International Patient Decision Aid Standards [29], can help to develop readable and comprehensible IC documents.

These different kinds of experts provide vital elements of IC documents. However, even multidisciplinary expert groups can hardly anticipate whether members of the target group will actually understand the given information and how they will assess the readability and comprehensibility of the IC documents [19, 30].

For this purpose, members of the target population should be involved in the process of testing and improving IC documents. Thus, in addition to the responsible researchers or medical experts and other professionals (ethicists, lawyers, and communication experts), prospective research participants and patients themselves can play an important role in the development of valid IC documents.

### Identifying and recruiting members of the target population

For meaningful involvement of members of the target population in the development of IC documents, it is essential to first identify a suitable target population. For certain kinds of research, the target population could be the general public. This applies, for example, to clinical studies designed to test new drugs in healthy humans, as well as for biobanks, which aim to collect and store samples from a particular region or population. However, the target population can also consist of patients suffering from a certain disease. Each kind of target population requires different recruitment methods.

Nevertheless, members of most target populations for research IC documents are lay persons, whose involvement in the evaluation and improvement of IC documents implies certain limitations. Although lay persons can express their own difficulties with reading and understanding the given information and their own emotional reactions or misunderstandings, they can hardly anticipate the reactions of other members of the target population, which could be very different from their own. Further, they usually lack knowledge of the development and conduct of clinical research, and are thus unable to identify missing information or mistakes in the explanations of certain facts. One possible way around these limitations is to involve ‘expert patients’ instead of or in addition to lay persons. Based on concepts of involving specially trained patients in the development and conduct of clinical research, the working definition of ‘expert patients’ as patient representatives who (1) are or were affected by an illness (directly or indirectly as a relative/proxy for children or cognitively-impaired adults) relevant to the disease under study, (2) have learned to engage in discussions about the disease from a patient standpoint and not only from their personal standpoint, and thus to anticipate the perspective of other patients, and (3) have in-depth knowledge about how to design and conduct clinical research, can be used [31, 32]. While property (1) also applies to ‘lay patients’, properties (2) and (3) require further knowledge and competencies. The concept of expert patients is rather new, and needs further development, so the potential benefits of involving expert patients in the development of IC documents remain unclear.

## Conceptual model for the development of understandable IC documents

On the basis of the conceptual and practical challenges outlined above, we propose an integrated model for the development, evaluation, and improvement of IC documents (Table 3). Many steps in the proposed model are already well developed, and have been applied successfully several times. This includes the use of mixed methods (questionnaires and semi-structured interviews) to assess the understandability of IC documents (e.g., [25, 26]), as well as randomized surveys to compare the quality of different versions of IC documents (e.g., [16, 33]). Other steps of the proposed model need more elaboration and investigation, e.g., the question of when and how to involve different experts or standards for clear writing in the IC development process, as well as the development of systematic strategies for revision of IC documents according to test readers’ feedback.

**Table 3:** Conceptual model for the development of understandable informed consent documents

Steps in the process of informed consent (IC) development	Action to take	Objectives
Writing of IC documents	Involve multidisciplinary expert groups in design of IC documents	− Ensure completeness and correctness of given information − Make sure legal and ethical requirements are met
	Involve communications experts and/or apply guidelines for how to design understandable written information	− Increase readability and understandability for lay people
Testing original IC documents	Identify the IC documents’ target population and develop strategies for recruiting test readers	− Make sure to recruit testers who are able to unveil or anticipate prospective research participants’ potential problems of understanding (depending on the IC documents’ actual target population, this could be members of the general public, lay patients, or expert patients) − Avoid systematic biases in groups of participants (e.g., according to education, age, sex)
	Clarify relevant concepts for testing: ‘understanding’	− Make testing results reliable and comparable to other testings (using the same concepts) − Adapt testing methods to applied concepts
	Quantitative element (questionnaire/quiz): test objective understanding; assess how easy it is to find and understand particular pieces of information	− Ensure the most important pieces of information are easy to find and understand − Take information from questionnaires for systematic preparation of focus groups or individual interviews
	Qualitative element (focus groups/individual interviews): assess subjective understanding, emotional reactions, and/or general understandability; discuss original IC documents with test readers	− Validate and complement results of questionnaires − Identify reasons for problems of understanding − Assess participants’ impression of completeness and balance of given information − Identify emotional reactions and misunderstandings − Learn about participants’ suggestions for improving IC documents
	Systematic summary of test readers’ feedback and suggestions	− Use as preparation for systematic revision − Identify consensual and conflicting opinions − Prioritize and organize potential changes in original document
Revising original documents	Develop rules to deal with different kinds of feedback	− Allow for systematic revision, not primarily based on authors’ experiences and personal taste
	Track revisions and explicitly link changes to feedback	− Make revisions and arguments for changes transparent and reasonable − Ensure changes actually address test readers’ needs and suggestions − Increase legitimacy of changes
	Involve original authors of IC documents and/or other experts	− Ensure the revised document still gives all relevant information − Ensure all given information is correct and meets legal and ethical requirements
	While making changes: apply guidelines for clear writing or involve communications experts	− Ensure standards for clear writing are met in revised version
Evaluating/re-testing revised documents	Quantitative element (questionnaire/quiz): test objective understanding for revised documents (as many iterations as necessary)	− Retest how easy to find and to understand most important pieces of information are in revised version − Take information from questionnaires for systematic preparation of focus groups or individual interviews − If necessary: revise and retest
	Qualitative element (focus groups/individual interviews): assess subjective understanding, emotional reactions, and/or general understandability for revised documents (as many iterations as necessary)	− Evaluate changes to original document − Ensure the most urgent needs and suggestions have been addressed − Validate revised version − Identify additional feedback and suggestions − If necessary: revise and retest
	Quantitative element: test final version against the original version by means of randomized survey	− Systematically evaluate the quality of the revised IC document in comparison to the original version

The proposed integrated model entails a rather sophisticated as well as costly process (with regard to both time and monetary resources). It may not be feasible for every single clinical research project to perform the whole model. However, at least parts of the model can be used in almost every research setting to validate and improve IC documents. Additionally, in certain research settings, template IC documents have been or are being developed, e.g., for clinical studies [34], by Research Ethics Boards for their addressees [35], or for biobanking in Germany [36]. For these templates, which will be used in several individual clinical research projects, it is vital that written information is understandable to prospective research participants and that it covers all their information needs. Thus, the above presented model for participatory IC improvement may be especially suitable for developing IC templates. Finally, some consent procedures entail particular ethical requirements and, therefore, justify a greater effort to validate and improve the quality of written information [36–39], e.g., when obtaining new forms of consent for biobank research or when inviting participants for studies involving high levels of risks or uncertainties such as in gene therapy or genome editing. During the development of IC documents for such forms of research, the above presented model for participatory consent improvement may also be an adequate means.

## Conclusions

There are a growing number of studies testing understanding and suggesting different measures to improve the consent process in this regard. However, several methodological and conceptual challenges remain unresolved in the assessment and improvement of understanding in IC. On the basis of our own experience as well as the relevant literature, we outlined an integrated conceptual model for the development, testing, and improvement of IC documents (Table 3). This rather sophisticated and time-consuming model is especially suitable for the development of IC templates that are going to be used in several individual studies, for the improvement of IC documents for clinical research involving particular risks and uncertainties (e.g., genome editing), or for IC documents obtaining broad consent (e.g., biobanks).

Further conceptual elaboration and empirical research is needed to continuously improve this model and to develop solutions to the unresolved challenges in the development, evaluation, and improvement of IC documents. What are the advantages and disadvantages of involving ‘expert patients’ compared to other potential test readers? How can IC documents be revised based on results from user testings or similar methods in a way that is sufficiently transparent and yet efficient?

To address the open research questions it might be fruitful, in future, for those involved in empirical evaluations of consent procedures to cooperate with those developing healthcare-related patient information.
